# Out of Africa: evidence of the obligate mutualism between long corolla tubed plant and long‐tongued fly in the Himalayas

**DOI:** 10.1002/ece3.1784

**Published:** 2015-10-22

**Authors:** Babu Ram Paudel, Mani Shrestha, Adrian G. Dyer, Xing‐Fu Zhu, Aysajan Abdusalam, Qing‐Jun Li

**Affiliations:** ^1^ Key Laboratory of Tropical Forest Ecology Xishuangbanna Tropical Botanical Garden Chinese Academy of Sciences Menglun Town Mengla County Yunnan 666303 China; ^2^ University of Chinese Academy of Sciences Beijing 100039 China; ^3^ Tribhuwan University Department of Botany Prithvi Narayan Campus Pokhara Nepal; ^4^ School of Media and Communication RMIT University Melbourne Victoria 3001 Australia; ^5^ Faculty of Information Technology Monash University Melbourne Victoria 3800 Australia; ^6^ Department of Physiology Monash University Clayton Melbourne Victoria 3800 Australia

**Keywords:** Alpine ginger, Nepal Himalayas, *Philoliche longirostris*, pollination, *Roscoea purpurea*, Tabanid fly

## Abstract

1. Mutualism between long corolla tubed plants and their potential pollinators, long‐tongued flies, is a classic example of coevolution, but to date, has only been reported from the regions of southern Africa. Many plant species from the Himalayas also show botanical characteristics that could be consistent with pollination by long‐tongued flies. Here, we seek the evidence of the “long‐tongued‐long tubed fly/flower” mutualism out of Africa, in a different continent and climatic region, the Himalayas.
2. Floral traits of Himalayan region endemic alpine genus, *Roscoea*, indicate possible mutualism with long‐tongued flies for pollination success; however, effective pollinators of this genus are yet unknown. This study investigates whether long‐tongued flies and *Roscoea purpurea* in Nepal Himalayas show exclusive mutualism for their survival/reproduction.
3. We made extensive observations of floral visitors of *R. purpurea* and food source of *Philoliche longirostris* across their wide ranges of populations in Nepal Himalayas for three consecutive years (2012–2014). To confirm the obligate reliance of *R. purpurea* upon *P. longirostris* for pollination success, manipulated pollination experiments were conducted at two populations for 2 years. Similarly foraging behavior, visitation frequency, and pollination efficiency of *P. longirostris* were assessed at two populations for 2 years, and its contribution for the reproductive success of *R. purpurea* was evaluated. Our results indicate that *R. purpurea* is self‐compatible but lacks autonomous selfing and obligatorily relies on *P. longirostris* for reproductive success. Across all populations, *P. longirostris* was observed as an exclusive and highly efficient pollinator of *R. purpurea,* while *P. longirostris* exclusively depends up on *R. purpurea* for food source.
4. Out of Africa, this study provides the first evidence of long‐tongued fly pollination system and indicates the possibility of additional instances of such a rare phenomenon in the Himalayas. Finding of specialized pollinator of *Roscoea* only at its evolutionary center indicates that *Roscoea* species are originally pollinated by long‐tongued flies. Spatial mismatch with specialized pollinators may have induced the evolution of autonomous selfing in North Indochinese clades of *Roscoea*. This finding thus substantiates how geographic disjunction causes the shifting of pollination mechanism in closely related plant species.

## Introduction

Relationship between long tubed flowers and their pollinators has gained insights since Darwin's (1862) prediction of long nectar spur Malagasy orchid (*Angraecum sesquipedale*) pollinated by long proboscid moth (Barrett 2010). Among the different pollinators such as birds, bats, moths, butterflies, and flies that contribute toward the pollination of long tubed plants, long‐tongued fly pollination system is a rare phenomenon that provides unique opportunity to study coevolution between the interacting traits of plants and pollinators (Goldblatt and Manning 2000; Krenn et al. 2005; Triponez et al. 2015). Although long proboscid flies are one of the foremost pollinators of angiosperms that coevolved concurrently with the flowering plants since late Jurassic (Labandeira 1998, 2010; Ren 1998), their role as pollinators has been recognized very recently (Marloth 1908; Vogel 1954). So far, this type of unique pollination system is known only from the Greater Cape Floristic region, Southern Africa (Goldblatt and Manning 2000). According to Goldblatt and Manning(2000), a total of fifteen species of flies belonging to family Nemestrinidae and Tabanidae having tongue longer than 15 mm are considered as long‐tongued flies, but all these species except one (*Philoliche longirostris* found in the Himalayas) have only been observed in Southern Africa. Although long‐tongued fly pollination system has attained considerable attention in Southern Africa (Goldblatt et al. 1995; Johnson and Steiner 1997; Manning and Goldblatt 1997; Goldblatt and Manning 1999; Johnson 2006; Combs and Pauw 2009), empirical evidence does not exist in other parts of the world. In the Himalayas, a couple of historic entomological observations (Fletcher and Son 1931; Dierl 1968) suggested the possibility that this rare pollination system may operate out of Africa.

The Himalayan region of Nepal is rich in biological diversity, and in both tropical and subalpine regions, flowers appear to confirm to general patterns of evolution for insect vision as observed in other regions of the world (Chittka and Menzel 1992; Dyer et al. 2012; Bischoff et al. 2013; Shrestha et al. 2014). However, at present the Himalayan region is relatively understudied compared to Europe (Chittka and Menzel 1992; Arnold et al. 2009), Africa (Johnson and Anderson 2010), and North America (McEwen and Vamosi 2010). Interestingly, Nepalese Himalayas include many plant species with long tubed flowers, belonging to different families such as Ericaceae, Geraniaceae, Scrophulariaceae, Orchidaceae, and Zingiberaceae that are presumed to be pollinated by long‐tongued insects (Vogel 1954; Goldblatt and Manning 2000). It is likely that *P. longirostris* visits some of these plants and is potentially an effective pollinator. While there are some observations of the long‐tongued fly *Corizoneura longirostris* was visiting the flowers of *Roscoea purpurea* (Zingiberaceae) a few decades ago (Fletcher and Son 1931; Dierl 1968), the recent status of this fly is unknown. Moreover, it is uncertain whether the fly is an important pollinator or just an occasional visitor to the flowers of *R. purpurea*. Thus, following the historical entomological observations of Fletcher and Son (1931) and Dierl (1968), we have selected *R. purpurea* as an experimental species to explore the possibility of long‐tongued fly pollination system in Nepalese Himalayas.

Africa is the center of evolution and/or diversification of ginger family (Zingiberaceae) and long‐tongued flies which evolved together since late Jurassic (Ren 1998; Grimaldi and Engel 2005; Kress and Specht 2006; Morita 2008). Because radiation of gingers and tabanid flies both occurred in late Cretaceous, similar to many other genera of plants and animals, their diversification toward the Indian plate is thus linked with Gondwana separation (Kress and Specht 2006; Lessard et al. 2013). Further radiation and speciation in Zingiberaceae, following the collision between Indian and Eurasian plate in the last 50 Ma, led to the evolution of long corolla tubed genus *Roscoea* from relatively shorter corolla tube genus *Hedychium* in the Himalayas (Ngamriabsakul et al. 2000; Zhao 2012; Zhao et al. 2015). Thus, we hypothesized that the evolution of *Roscoea* is selected by the long‐tongued flies that diverged parallel with the ancestors of *Roscoea*, while the existence of *P. longirostris* in the Himalayas also depends upon *Roscoea* species as a key food source. Hence, in this study, we investigated the mutual reliance of long‐tongued fly (*P. longirostris*) and long corolla tubed alpine ginger (*R. purpurea*) in Nepal Himalayas.

The genus *Roscoea*, unlike other members of ginger family that are distributed in tropical and subtropical habitats, is an alpine ginger group endemic to the Himalayan regions (Cowley 1982, 2007). It comprises of two disjunct groups (Himalayan and North Indochinese clades) separated by a topographic barrier of ~500 km (Cowley 2007; Zhao et al. 2015). The two groups split into distinct lineages at ~23 (13–38) Ma with the rapid lateral extrusion of Indochina (Zhao et al. 2015). Most species of *Roscoea* have several features that have previously been implicated in long‐tongued fly pollination syndromes such as bright colored zygomorphic flower, wide labellum as a landing platform to its pollinators, nectar filled long corolla tube, elongated anther and stigma located far from nectar's source, and absence of discernible fragrance (Lynch 1882; Goldblatt and Manning 2000; Fenster et al. 2004). However, recent studies on the reproductive biology of North Indochinese clades of *Roscoea* did not find any effective pollinators as predicted by the floral syndrome and these species either reproduce by autonomous selfing or are pollinated occasionally by generalist pollinators (Zhang and Li 2008; Zhang et al. 2010; Fan and Li 2012). Zhang et al. (2010) predicted that original pollinators of *Roscoea* were long‐tongued insects, which have been lost from North Indochina due to recent climate change that occurred in the Chinese Himalayas. Although long‐tongued fly is apparently lost from the Chinese Himalayas, we anticipated that this fly still exists in the Nepal Himalayas and potentially be an effective pollinator of long corolla tubed *R. purpurea*. Thus, in this study, we broadly investigated the floral visitors of *R. purpurea* and behavior of *P. longirostris,* evaluated the pollination efficiency of *P. longirostris,* and conducted artificial pollination experiments to address the following questions (1) How does *R. purpurea* reproduce in natural condition? (2) Does long‐tongued fly act as effective pollinator of this alpine ginger in Nepal Himalayas? (3) Do *R. purpurea* and *P. longirostris* show obligate mutualism for their co‐occurrence, survival and/or reproduction?

## Materials and Methods

### Study species and sites

*Roscoea purpurea* is a perennial herb growing in both damp and dry subalpine to alpine slopes; between the elevation of 1520 and 3100 m asl (Cowley 1982, 2007). Plants are usually 20–60 cm tall, with annual leafy shoot produced from erect rhizomes. Lowermost leaf is slightly auriculate and rest of the leaves are not auriculate. Flowering occurs from late June to September. Inflorescence is enclosed in upper leaf sheath. Flowers are usually purple in color. Apex of bracts exserted above leaves and is longer than calyx. Spathulate staminodes are white or mauve veined and 2.5–4 × 0.6–1.1 cm in dimensions. Anther appendages are 9–25 mm long. Anthers and pistil are white in color (Cowley 1982, 2007).

**Figure 1:**
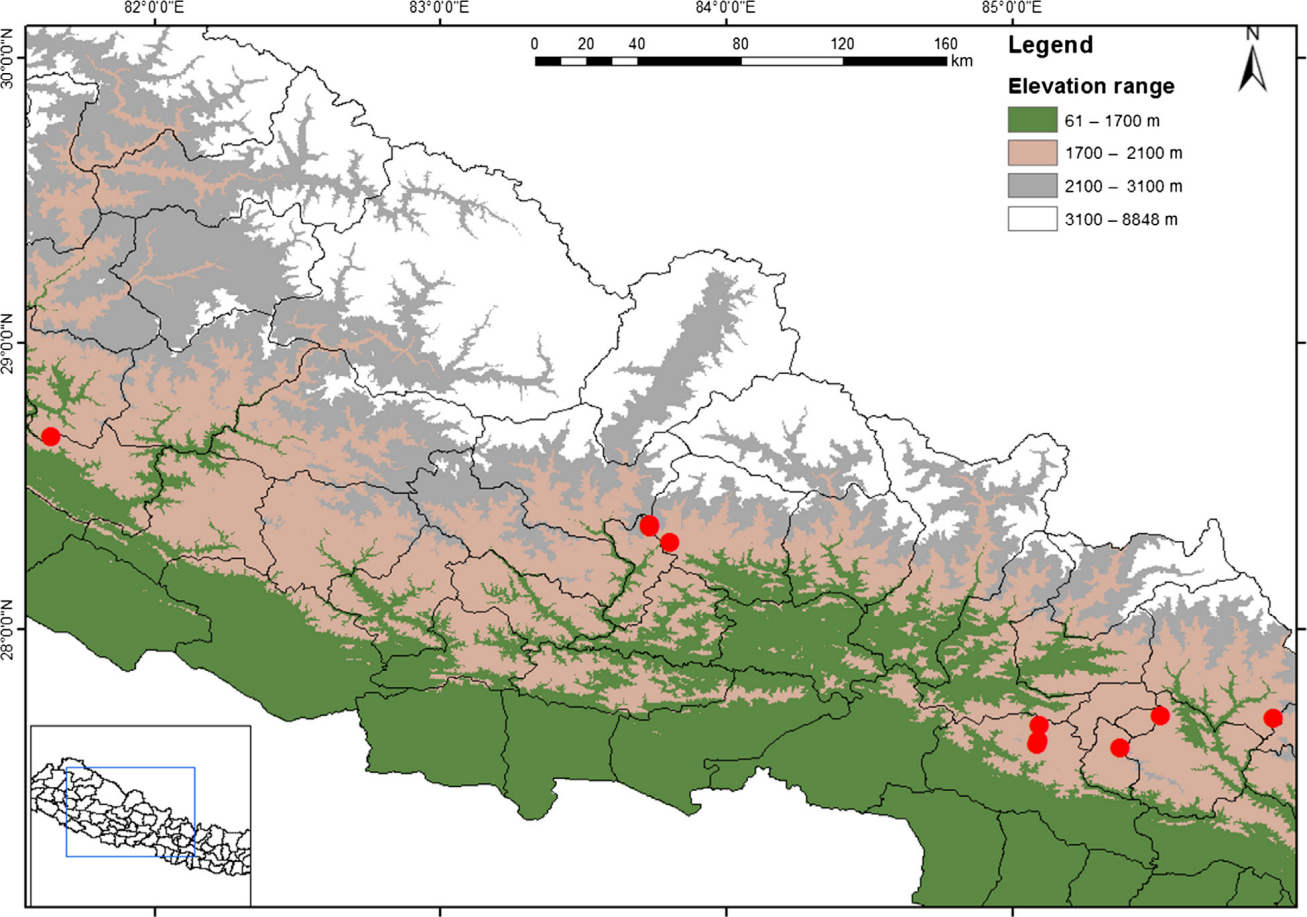
Map of experimental sites in Nepal Himalaya.

Manipulated pollination experiments were performed for two consecutive years from July to September (2013 and 2014) at Daman (27°36′35.7″ N, 85°05′37.3″ E; altitude 2319 m) and Mudhe (27°41′11.0″ N, 85°55′2.6″ E; altitude 2453 m), Central Nepal. At both sites, large numbers of individuals were growing under the canopy of *Pinus* and *Rhododendron* forest. While floral visitors of *R. purpurea* were observed across eight additional populations that represent the distribution ranges of *R. purpurea* along different elevation gradients in Nepal Himalayas. Study sites and their geographical details are presented in Figure 1 and Table S1.

### Floral biology

We recorded the floral biology of *R. purpurea* at Daman and Mudhe from early July to late August (2013 and 2014). Twenty flowering individuals at each population were randomly selected, and number of inflorescence per plant and number of flower or bud per inflorescence were counted. Floral longevity, time of anthesis, time of anther dehiscence, length of corolla tube, length of ovary, and length of anther were measured from freshly opened flowers (*n* = 20), following the method of Fan and Li (2012). As the hollow cavity of corolla tube was too small to accommodate micro‐capillaries, we were unable to measure the nectar volume. We thus estimated the volume of nectar by backlighting the corolla tube following the method of Alexandersson and Johnson (2002). For pollen and ovule count, 20 matured buds at each site were randomly selected and fixed in 70% ethanol. Following the method of Dafni (1992), the number of pollen grains per flower and ovules in each ovary was counted with a microscope. Pollen–ovule ratio (P/O) of each flower was calculated as the number of pollen grains divided by the number of ovules. For all the floral characters, mean value with standard errors was computed. We used independent sample *t*‐test to examine the differences in floral traits between two sites.

### Observation of floral visitors

We observed both diurnal and nocturnal insects that visit the flowers of *R. purpurea*, across ten populations (Table S1). For diurnal visitors, observations were made from 7.00 am to 19.00 pm, while nocturnal visitors were observed from 19.00 pm to 21.00 pm with the help of a flashlight covered with a red plastic. However, detailed study of the behavior of *P. longirostris* (an obligate visitor of *R. purpurea*) was performed only at two sites (Daman and Mudhe). We made two plots (10 m × 10 m) in each site, at the patches where plants were densely flowered. The distances between two plots were maintained greater than 100 m. At each plot, observations were made continuously for 4 h with two observation periods each day. Observations were repeated for three consecutive days, at each respective plot and site. Visitation frequency and flower foraging time of *P. longirostris*, for the intervals of every hour, were recorded throughout the day. Visitation frequency was calculated in terms of number of visit flower^−1 ^h^−1^. Foraging time was calculated by measuring the time spent by a fly in a flower. The foraging behavior of fly was recorded through direct observation. To test the effect of population and year on visitation frequency and foraging time of the fly, a two‐way ANOVA was computed with population and year as fixed factors. To determine the nectar accessibility, tongue length of the fly and the distance from the entrance of corolla tube to the top level of nectar were measured using digital vernier caliper.

### Pollination efficiency of fly

Following Gross (2005) and Néeman et al.(2010), we have adopted two methods (stigmatic pollen deposition method and reproductive success method) to calculate the pollination efficiency of *P. longirostris*. At each site, 40 matured buds of *R. purpurea* were randomly selected and covered by mesh bags. On the subsequent morning, the bags were removed and flowers were thus exposed to the pollinators. When a fly made a single visit to a virgin experimental flower, either the flower was immediately covered by a mesh bag to exclude other pollinators or the stigma of the flower was carefully collected and fixed in 70% alcohol. Among the 40 experimental flowers foraged by a fly in its single visit, stigmas were collected from 20 flowers and next 20 flowers were immediately covered by mesh bags. When those flowers foraged by a fly subsequently wilted, the mesh bags were removed. On the other hand, all the collected stigma samples were transported to laboratory and pollen grains deposited on each stigma were counted under a microscope at 40× following the method of Dafni (1992). Pollination efficiency of a fly by stigmatic pollen deposition method was calculated following Inouye et al.(1994). The overall pollination effectiveness of a fly was calculated by multiplying the stigma pollen load and visitation frequency (Fumero‐Caban and Melendez‐Ackerman 2007). For estimating pollination efficiency of a fly by reproductive success method, fruit set percentage of those experimental flowers were calculated and seed numbers per fruit were counted (Inouye et al. 1994; Néeman et al. 2010).

### Pollination treatments

To determine the natural breeding system of *R. purpurea*, a single bud from 240 flowering individuals, from within a plot of 20 m × 20 m, was randomly selected and covered by mesh bags until anthesis occurred. Those experimental flowers were then randomly assigned into six pollination treatment groups including (1) open pollination (control); (2) pollinators exclusion without emasculation (autonomous selfing); (3) pollinator exclusion with emasculation (apomixis); (4) hand self‐pollination – flowers were hand self‐pollinated using the pollen grains of same flowers and covered by fine mesh bag to exclude pollinators; (5) hand cross‐pollination – flowers were first emasculated and then hand cross‐pollinated using the pollen grains of other individuals lying more than 5 m away and covered by fine mesh bag to exclude pollinators; and (6) supplementary pollination flowers were exposed for natural pollination and continuously supplemented by foreign pollen grains until the flowers wilted. All the mesh bags were removed when flowers wilted. We collected the fruits from each treatment separately after about 30 days, and number of fruit set and seed per fruit were counted. Percentage of fruit set was calculated as the ratio of number of fruits to the number of flowers. To determine the effect of population on the reproductive mechanism of *R. purpurea*, manipulated pollination treatments were conducted at two sites (Daman and Mudhe) in 2013. Pollination treatments (1) and (6) were repeated at both sites in 2014 also, to examine the effect of year on pollen limitation.

Differences in fruit set percentage and seed number per fruit between hand self‐pollination and hand cross‐pollination were analyzed using generalized linear model (GLM) with binomial and Poisson distribution of errors, respectively. In GLM, sites and treatments were used as fixed factors. Self‐incompatibility indices (SI) at each site were calculated following Ruiz‐Zapata and Arroyo (1978). To examine the effect of year, sites and treatments in fruit set percentage, and seed number per fruit between natural and supplementary pollination, we used GLM with binary and Poisson distribution of errors, respectively. Pollen limitation indices at each site and year were calculated following Oliveira and Gibbs (2000).

## Results

### Floral biology

Flowering in *R. purpurea* occurred from late June to early September with peak flowering from late July to early August, at both sites. An individual plant, at both sites, produced a single inflorescence and each inflorescence contained 6–17 flowers (*n* = 40). Anthesis occurred in early morning and soon the anther became ready for dehiscence. Flowering within an inflorescence proceeded in centripetal manner. A single flower survived 4–7 days (*n* = 40) and whole inflorescence survived 20–30 days. Except corolla tube length, number of pollen grains and pollen–ovule ratio (P/O value), the remaining floral characters did not differ significantly between two populations (*P *>* *0.05, Table 1).

**Table 1:** Floral characters of *Roscoea purpurea*. Mean (±SE) of floral characters of *R. purpurea* and tongue length of *Philoliche longirostris* at Daman and Mudhe (independent sample *t*‐test, *N* = 20). Bold *P* values represent significant difference at *P* < 0.001

Floral characters	Daman	Mudhe	*t*‐test	*P* value
No. of flowers/inflorescence (*n*)	9.60 ± 0.78	8.40 ± 0.63	1.134	0.271
Floral longevity (days)	5.10 ± 0.18	4.95 ± 0.16	0.645	0.527
Length of corolla tube (mm)	89.55 ± 1.06	72.3 ± 1.16	10.243	**0.000**
Nectar distance from the entrance of corolla tube (mm)	33.90 ± 0.96	34.25 ± 1.00	−0.237	0.815
Tongue length of *P. longirostris*	58.8 ± 1.37	40.7 ± 0.69	11.87	**0.000**
Nectar level from top of ovary (mm)	55.65 ± 1.55	54.25 ± 1.54	0.656	0.519
Anther's length	8.20 ± 0.19	8.35 ± 0.20	−0.616	0.545
Length of ovary	17.85 ± 0.60	17.70 ± 0.50	0.196	0.847
No. of pollen grains/flower	20668.50 ± 1545.58	7372.00 ± 647.18	6.943	**0.000**
No. of ovules/flower	45.95 ± 2.12	43.35 ± 1.077	1.264	0.222
Pollen–Ovule ratio (P:O)	476.18 ± 47.03	172.06 ± 15.62	5.563	**0.000**

### Observation of floral visitors

**Figure 2:**
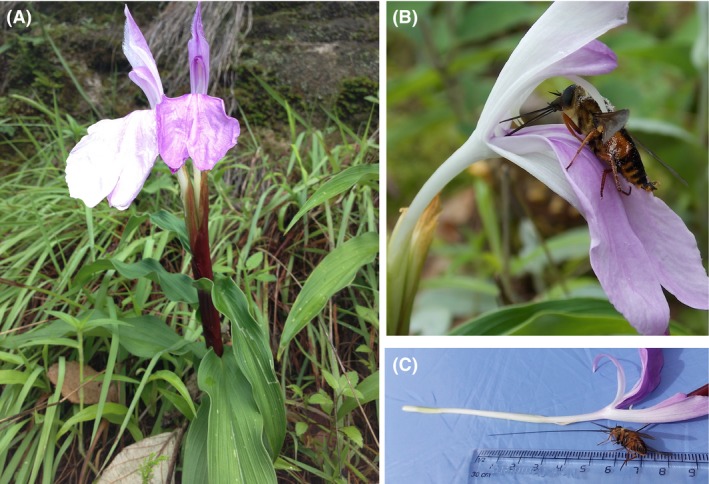
Study organisms. (A) – A flowering individual of *Roscoea purpurea* in its natural habitat, (B) – *Philoliche longirostris*, an obligate pollinator of *R. purpurea* sipping nectar from the flower of *R. purpurea*, (C) – compatibility between nectar distance in the corolla tube of *R. purpurea* and tongue length of *P. longirostris*.

**Figure 3:**
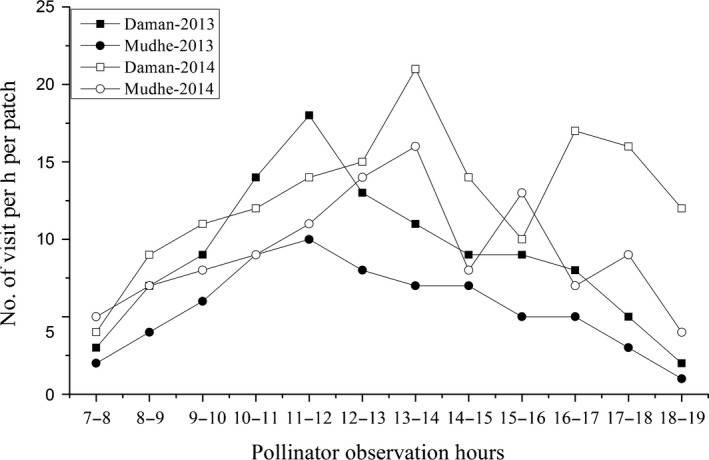
Hourly variation in frequency of *Philoliche longirostris* visiting the flowers of *Roscoea purpurea* from 7:00 am to 19:00 pm at two sites and two years.

A long‐tongued fly (*Philoliche longirostris*, Fig. 2B) was observed as an exclusive diurnal floral visitor of *R. purpurea* across all ten sites. But we did not observe any nocturnal visitors throughout all sites. The fly showed constancy in the floral visit to *R. purpurea* and did not show any evidence of searching for the flowers of other species that coflower with *R. purpurea* (see Movie S1, supporting information). Our observations showed that the fly first landed on the labellum, and then, its activity proceeded as follows: The fly slightly moved a little up toward the mouth of corolla tube and inserted its tongue inside the corolla tube to sip the nectar. By this time, the fore legs and head region of the fly pressed the lever like anther appendages. This action caused the filament to bend forward and thus the anther lobe and stigma touched the back of the fly. This eventually led to the deposition of large number of pollen grains on the back of fly while stigma received pollen grains already carried by the fly from other flowers (Fig. 2B). The activity of flies increased in sunny hours and decreased in early morning, late evening (Fig. 3) and during cloud cover, but the flies did not visit the flowers if the rain was present.

Visitation frequency of *P. longirostris* differed significantly between years (*P *<* *0.001) and interaction of years and populations (*P *<* *0.05) but did not differ significantly between populations (*P *>* *0.05, Table S2). Visitation frequency of fly was highest at Daman in 2014 and least at Daman in 2013 (Figure S1). Foraging time did not differ significantly across years, populations, and their interactions (*P *>* *0.05, Table S2). At both sites, average tongue length of *P. longirostris* was shorter than average corolla tube length of *R. purpurea*; however, tongue length was long enough to reach the nectar (Table 1, Fig. 2C).

### Pollination efficiency of fly

**Figure 4:**
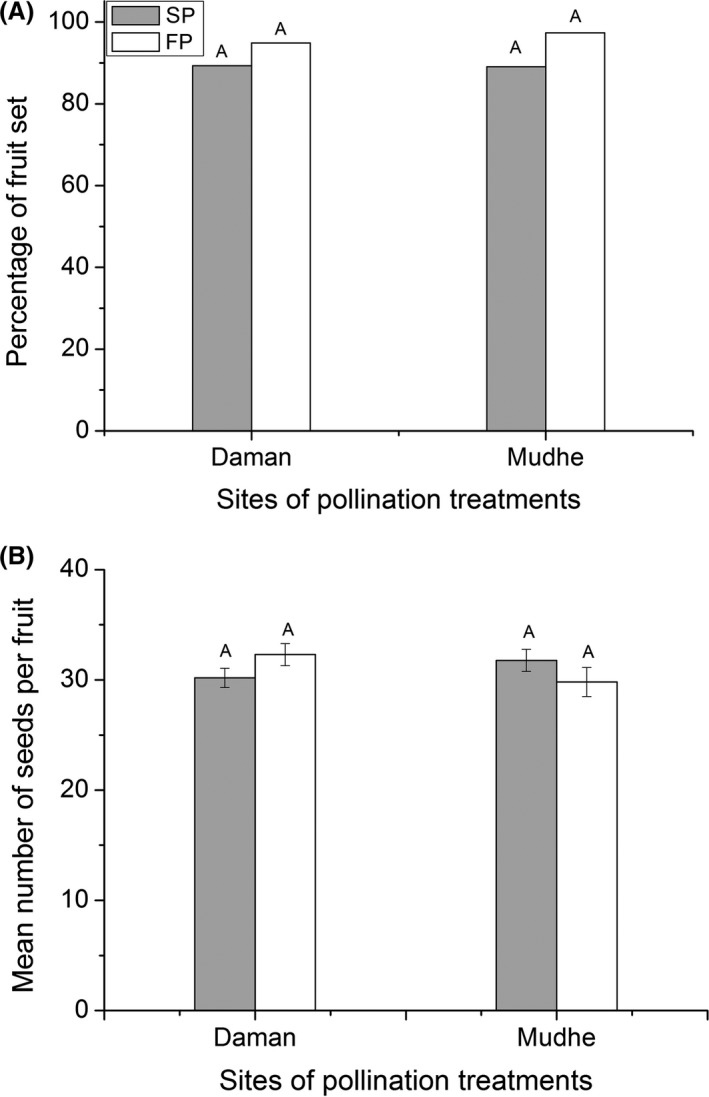
Pollination efficiency of *Philoliche longirostris*. (A) Mean fruit set percentage and (B) Mean seed number per fruit between supplemented and fly pollinated (upon a single visit) flowers of *Roscoea purpurea* at two sites (Daman and Mudhe). SP and FP represent supplemental and fly pollination respectively.

Mean number of pollen grains deposited on a virgin stigma and pollination efficiency indices of *P. longirostris* at two sites did not differ significantly (*t*‐test, *P *>* *0.05, see Table S3, supporting information). At both sites, number of pollen grains deposited on a stigma was many times larger than the number of ovules per flowers. Fruit set percentage and seed number per fruit, formed upon a single visit of a fly, at two sites did not differ significantly (*t*‐test, *P *>* *0.05, see Table S3, supporting information). At both sites, a large proportion of flowers (94.9% at Daman and 97.4% at Mudhe) visited by the fly set fruit. This indicated that single visit by a fly was sufficient for pollination success in *R. purpurea*. At both sites, and in both years, fruit set percentage and seed number per fruit in fly pollinated flowers were greater than in supplemental pollination (Fig. 4), but did not differ significantly (*P *>* *0.05). This result showed that *P. longirostris* is highly efficient pollinator of *R. purpurea*.

### Breeding system

Among the six pollination treatments, control + bagged and emasculated + bagged flowers at respective sites and years did not set the fruit. However, open flowers and three types of hand‐pollinated (self, cross, and supplemented) flowers set fruit and seed. Percentage of fruit set between hand self‐pollinated and hand cross‐pollinated flowers did not differ significantly between populations, treatments, and their interaction (*P *>* *0.05, see Table S4 and Figure S2, supporting information). Seed number per fruit differed significantly between treatments but did not differ significantly between populations and the interaction of population and treatment (see Table S4 and Figure S2, supporting information). Mean values of self‐incompatibility indices were greater than 0.5 at both sites. This result shows that *R. purpurea* is highly self‐compatible (Ruiz‐Zapata and Arroyo 1978).

**Figure 5:**
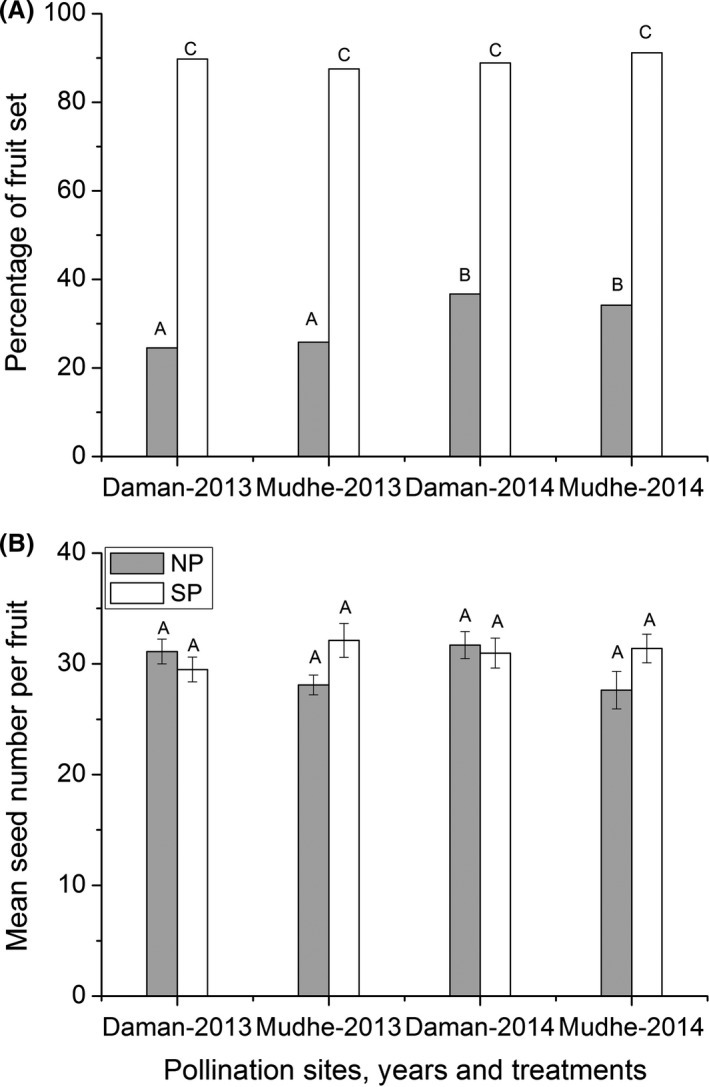
Pollen limitation in *Roscoea purpurea*: Fruit set and seed set by natural and supplemented pollination at two sites and years. (A) Percentage of fruit set between natural NP and supplemented (SP) pollination. (B) Number of seeds per fruit of NP and SP treatments. Error bars represent standard error of mean. Different letters indicate significant statistical difference at *P* < 0.05.

Open pollinated flowers, at both sites and years, set significantly lower percentage of fruit than the flowers pollinated by supplemental treatments (Fig. 5A). Fruit set percentage of these two treatments differed significantly between years and treatments (*P* < 0.05, see Table S5, supporting information), but not between populations and all interactions of year, populations, and treatments (*P* > 0.05, see Table S5, supporting information). Natural fruit set percentage differed significantly while supplementary fruit set did not differ significantly between years (Fig. 5A). Seed number per fruit of these two treatments did not differ significantly between years, populations, treatments (Fig. 5B), and all their interactions except the interaction between population and treatment (see Table S5, supporting information). At both sites and years, pollen limitation indices (PLI) at fruit set level were relatively higher than PLI at seed set stage.

## Discussion

### Specialized pollination system

Our result shows that *R. purpurea* is not capable of autonomous self‐pollination and apomixis, and completely relies on pollinators for pollination success. Obligate reliance of this alpine ginger upon a single pollinator (*P. longirostris*) thus indicates highly specialized pollination system. Our 3 years of field observation across ten populations indicated *P. longirostris* as an obligate pollinator of *R. purpurea*. Extremely high pollination efficiency of *P. longirostris* and synchronized blooming period of plant with the winged stage of fly (personal observation) further supports that this fly is the principal pollinator of *R. purpurea*. Although bumblebees and moths were active during the blooming period of *R. purpurea*, they only approached near the flower but aborted the flight and did not land on flowers of *R. purpurea*. Our result shows that nectar level in *R. purpurea* is situated deep inside the corolla tube (>34 mm) but tongue lengths of bumblebees and moths were shorter than nectar distance. The mismatch between their tongue length and nectar distance may be a reason why bumblebees and moths choose to abort the flowers of *R. purpurea*. Thus, *R. purpurea* exhibits specialized pollination system and avoids its noneffective visitors through elongation of corolla tube. Further, the frequent visits of long‐tongued flies may reduce nectar level in the corolla tube, and thus, nectar becomes inaccessible to other nonspecific pollinators. This result is inconsistent with the results of previous researches on two *Roscoea* species in China, which lack effective pollinators and reproduce by autonomous selfing (Zhang and Li 2008; Fan and Li 2012). However, self‐compatible nature of this species is consistent with *R. schneideriana*,* R. cautleoides*, and *R. debilis* (Zhang and Li 2008; Zhang et al. 2010; Fan and Li 2012).

### Floral longevity and pollen limitation

In this study, we have found that natural fruit set in *R. purpurea* is severely affected by pollen limitation, but seed set has no effect of pollen limitation. This indicates that low percentage of fruit set in natural population of *R. purpurea* is due to insufficient abundance of pollinators rather than insufficient deposition of pollen grains by a pollinator during a flower visitation. Thus, like other alpine plants (Bingham and Orthner 1998; Ashman et al. 2004), natural populations of *R. purpurea* suffered from pollinator limitation. Previous studies have shown that increased floral longevity is one of the reproductive strategies of alpine plants to ensure reproductive success in a zone of unpredictable pollinator abundance (Bingham and Orthner 1998; Blionis et al. 2001; Rathcke 2003). Our present result shows that flowers of *R. purpurea* have relatively longer life span (average 4.95 ± 0.16 to 5.10 ± 0.18; *n* = 40), unlike other members of Zingiberaceae which have usually 1 day floral longevity (Larsen et al. 1998 but see Zhang et al. 2010). Longer floral life span of *R. purpurea* may thus ensure higher pollination success by increasing the probability and frequency of visiting a flower by a pollinator (Primack 1985; Ashman and Schoen 1994). Zhang et al. (2010) also found that increased floral longevity is the unique reproductive strategy of *R. cautleoides* and *R*. *humeana* to tackle with the low frequency of nonspecific pollinators. On the contrary, selfing species of same genus *R. debilis* have relatively shorter floral longevity (3 days) (Fan and Li 2012). Hence, it can be speculated that increased floral longevity has enabled *R. purpurea* to cope with low densities of pollinators and ensure pollination success.

### The original pollinator of *Roscoea*

Gingers and long‐tongued tabanid flies are the Gondwanan lineages that evolved simultaneously in late Jurassic, synchronous with the supercontinent Pangaea separation, and are one of the earliest angiosperms/pollinators (Ren 1998; Grimaldi and Engel 2005; Kress and Specht 2006). Following the fragmentation of historical landmass (Gondwana), gingers and fly (*Philoliche*) both showed parallel diversification trajectory toward the Indian plate from their ancestral distribution center (Africa) (Kress and Specht 2006; Morita 2008; Lessard et al. 2013). A molecular phylogenetic analysis based on nuclear and chloroplast genome of 17 species of *Roscoea* showed that this monophyletic genus was evolved in the Himalayas following the Indian collision with Eurasian at ~50 Ma (Zhao 2012; Zhao et al. 2015). Among 22 *Roscoea* species currently distributed in Himalayas and North Indochina, *R. purpurea* is one of the oldest (ancestral) species of this genus (Cowley 2007; Zhao 2012). Based on our current finding of *P. longirostris* as an obligate and specialized pollinator of *R. purpurea*, it can be speculated that the evolution of *Roscoea* was selected by the long‐tongued flies and most probably by *P. longirostris* because this is the only species of long‐tongued flies that exists in the Himalayas (Goldblatt and Manning 2000).

Our finding of *P. longirostris* as a specialized pollinator of *R. purpurea* in Nepal Himalayas but its absence in southwest China has led us to hypothesize two possible consequences. (1) When the genus *Roscoea* split into two distinct lineages (Himalayan and North Indochinese clades) as a consequences of the third uplift of the Himalayas (Zhao et al. 2015), this topographic movement may have formed a geographic disjunction for the diversification of long‐tongued fly toward the Chinese Himalayas. Thus, this created the spatial mismatch between Chinese *Roscoea* and their effective pollinators, and hence in the absence of specialized pollinator, they may have evolved autonomous selfing mechanism to ensure reproductive success. Indeed, it has been suggested (Hegland et al. 2009) that spatial or temporal mismatch in pollinators and plants may lead to the evolution different pollination systems under climate change scenarios, and the natural steep terrain of the Himalayas can provide important insights into how such effects have driven plant–pollinator interactions in the past. (2) Long‐tongued flies were lost from the Chinese Himalayas as a consequence of recent climate change associated with the tectonic movement. With the subduction of Indian plate below the Eurasian Plate, lateral extrusion occurred in Indochina which caused 90° clockwise rotation in eastern part of Himalayan–Tibetan plateau (Zhao et al. 2015). This geological movement thus changed the east–west orientation of Chinese Mountains into north–south. Thus, the fragile environment of Chinese Himalayas associated with north–south Mountains and valleys may have been hostile for the fly that requires high relative humidity and temperature to complete its life cycle (Baldacchino et al. 2014). Hence, the fly may have become extinct from the Chinese Himalayas. Zhang et al. (2010) also predicted that long‐tongued insects (the original pollinators of *Roscoea*) are lost from the Chinese Himalayas due to recent climate change associated with the uplift of the Himalayas and lateral extrusion of Indochina. Nevertheless, extensive observations are needed to address why long‐tongued fly is absent in the Chinese Himalayas as the global climate change is causing pollinators decline in many parts of the world (Potts et al. 2010).

### The first evidence of long‐tongued fly pollination system in Himalayas

Long‐tongued fly pollination system is fairly uncommon phenomenon first observed by Marloth (1908) and then subsequently elaborated by Vogel (1954) from southern Africa. Since 1990, considerably large number of studies on long‐tongued fly pollination system have been documented; but such studies have been confined to Southern Africa (Goldblatt and Manning 2000). Out of Africa, our result provides the first experimental evidence of long‐tongued fly pollination system in a different continent and climatic region, the Himalayas. Although such a specialized mutualism between long‐tongued flies and the plants they pollinate forms a small portion of global biodiversity, it has been used as a model system to study pollinator mediated selection and coevolution (Johnson and Steiner 1997; Pauw 2007; Anderson and Johnson 2009; Pauw et al. 2009). Our result showed that corolla tube length of *R. purpurea* and tongue length of *P. longirostris* varied concordantly at two sites. This may indicate possible coevolution between these interacting traits. We anticipate that this finding will promote further research to explore the possible coevolution between tongue length of *P. longirostris* and corolla tube length of *R. purpurea*. It is also likely that *P. longirostris* may be involved as an agent of phenotypic selection on floral traits of *R. purpurea* and other Himalayan plants such as other species of *Roscoea*, Orchids, *Rhododendron*, which also show botanical characteristics that could be consistent with pollination by long‐tongued flies (Goldblatt and Manning 2000). We are planning for further research to explore the additional instances of long‐tongued fly pollination system and pollinator mediated selection in the Nepalese Himalayas, including more genera of plants and flies.

### Implication for conservation

Collapse of obligate mutualism between plant and pollinator is one of the causes of global biodiversity loss (Bond 1994). Our result showed that *P. longirostris* is a keystone pollinator of *R. purpurea* and in turn *R. purpurea* is the only known food source for the fly. Synchronized overlap between winged stage of *P. longirostris* and blooming period of *R. purpurea* indicates their exclusive reliance upon each other for existence and/or reproduction. Although the fly needs sufficient nectar to fulfill its daily requirement (Johnson 2006), our observation did not find any alternative long corolla tubed plants that coflower with *R. purpurea,* which may serve as additional food sources to the fly. Other species of *Roscoea* and some species of *Rhododendron* and Orchids are likely to be the additional food sources of this fly, but their blooming period is quite earlier than the winged stage of fly (personal observation). On the other hand, in the absence of any alternative pollinator, *R. purpurea* also possess severe risk of reproductive failure because this species entirely relies on pollinator for reproductive success. Like other tabanid flies, this fly also has complex life cycle and requires wetland habitat for larval development, while female adult requires blood meal to develop eggs (Lehane 2005). Thus, in addition to sufficient food sources, an undisturbed area with aquatic ecosystem and a suitable mammal host are required to complete the life cycle of *P. longirostris* (Goldblatt and Manning 2000; Lehane 2005; Baldacchino et al. 2014). Hence, these factors are potentially necessary to be incorporated to implement conservation strategy for the highly specialized mutualism between closely dependent plant and animal, like *R. purpurea* and *P. longirostris*.

## Conclusion

Out of Africa, this study provides the first empirical evidence of the obligate mutualism between long‐tongued fly and long corolla tubed plants for their survival/reproduction and highlights the needs of further researches to explore additional evidences of such a unique and rare mutualism in the Himalayas. This African originated unique plant–animal mutualism found in the Himalayas enhances the biogeographic relationship between South Asia and Africa in origin and diversification of biodiversity via Indian collision with Eurasian at ~50 Ma. The finding of *P. longirostris* as an obligate and extremely specialized pollinator of *Roscoea* at its evolutionary center, which is not found in secondary habitat of *Roscoea*, indicates that selection by long‐tongued fly is one of the major factors of evolution/speciation of *Roscoea* in the Himalayas. Meanwhile, the finding of contrasting pollination mechanism between Himalayan and North Indochinese clades of *Roscoea* substantiates how spatial mismatch between plants and their specialized pollinators induces shift of pollination mechanism in closely related plant species. Moreover, the absence of *P. longirostris* in the Chinese Himalayas contributes to understand how topographic movement, geographic disjunction, and climate fluctuation affect the diversification/existence of plants and pollinators. In the context of present global climate change, it is likely that the fly may have become extinct from the Chinese Himalayas. Thus, it is very essential to know how the behavior of native plant and pollinator is influenced by the global climate change and indicates the emergency of conservation efforts.

## Conflict of Interest

None declared.

## Supporting information


**Table S1**. Geographical details of study sites.
**Table S2**. Variation in the behavior of *P. longirostris* across sites and years.
**Table S3**. Pollination efficiency of *P. longirostris* at two sites.
**Table S4**. Test of self‐compatibility in *R. purpurea*.
**Table S5**. Test of pollen limitation in *R. purpurea*.
**Figure S1**. Variation in visitation frequency and foraging time of *P. longirostris* in the flowers of *R. purpurea* between sites and years.
**Figure S2**. Evidence of self‐compatibility in *R. purpurea*.

**Movie S1**. Foraging behavior of *P. longirostris* upon the flowers of *R. purpurea*.
