# Supplementary material for: Out of Africa: evidence of the obligate mutualism between long corolla tubed plant and long‐tongued fly in the Himalayas
Source: Ecol Evol. 2015 Oct 22;5(22):5240–51. doi: 10.1002/ece3.1784 (PMC6102519; doi:10.1002/ece3.1784)
Supplement: Supplementary file 1 — Table S1. Geographical details of study sites. Table S2. Variation in the behavior of P. longirostris across sites and years. Table S3. Pollination efficiency of P. longirostris at two sites. Table S4. Test of self‐compatibility in R. purpurea. Table S5. Test of pollen limitation in R. purpurea. Figure S1. Variation in visitation frequency and foraging time of P. longirostris in the flowers of R. purpurea between sites and years. Figure S2. Evidence of self‐compatibility in R. purpurea. [file ECE3-5-5240-s001.docx]

## **Supporting information**

**Table S1:** Geographical details of study sites. Manipulated pollination experimental sites are indicated by an asterisk mark.

| SN | Name of sites | Latitude | Longitude | Altitude |
| --- | --- | --- | --- | --- |
| 1 | Phulchoki | 27°34'55.6'' | 85°22'59.3'' | 1903 m |
| 2 | Lumle | 28°18'14.4'' | 83°48'16.3'' | 1957 m |
| 3 | Tistung | 27° 39′ 36.28″ | 85° 5′ 58.07″ | 2014 m |
| 4 | Ulleri | 28°21'24.44'' | 83°44'8.48'' | 2039 m |
| 5 | Nagarkot | 27°41'39.1'' | 85°31'16.1'' | 2117 m |
| 6 | Ranimatta | 28°40'18.3'' | 81°38'18'' | 2158 m |
| 7 | Banthati | 28°22'0.1'' | 83°44'1.4'' | 2222 m |
| 8 | Daman* | 27°36'32.8'' | 85°5'36.6'' | 2334 m |
| 9 | Mudhe* | 27°41'13.6'' | 85°55'1.1'' | 2435 m |
| 10 | Simbhangjyang | 27°35'37.4'' | 85°5'17.3'' | 2574 m |

**Table S2: Variation in the behaviour of *P. longirostris* across sites and years**. Result of two way ANOVA showing the effect of years and populations on visitation frequency and foraging time *P. longirostris* to the flowers of *R. purpurea.*

| Source | Sum of Squares | df | Mean Square | F | P |
| --- | --- | --- | --- | --- | --- |
| **Visitation frequency(visit/fl/hr)** |  |  |  |  |  |
| Years | 0.196 | 1 | 0.196 | 36.355 | **0.000** |
| Populations | 0.017 | 1 | 0.017 | 3.115 | 0.085 |
| Years x Populations | 0.040 | 1 | 0.040 | 7.347 | **0.010** |
| Error | 0.237 | 44 | 0.005 |  |  |
| **Foraging time (s)** |  |  |  |  |  |
| Years | 0.174 | 1 | 0.174 | 0.097 | 0.757 |
| Populations | 0.296 | 1 | 0.296 | 0.165 | 0.687 |
| Years x Populations | 2.007 | 1 | 2.007 | 1.118 | 0.296 |
| Error | 78.966 | 44 | 1.795 |  |  |

**Table S3: Variation in pollination efficiency of *P. longirostris* across sites.**  Result of independent sample t test between number of pollen grains deposited on a virgin stigma, pollination efficiency index, percentage of fruit set and number of seed/fruit formed upon a single visit by a fly to the flowers of *R. purpurea* at two populations.

|  | Daman | Mudhe | t test | P value |
| --- | --- | --- | --- | --- |
| Number of pollens deposited | 1236.12±51.65 | 1177.78±67.09 | 0.629 | 0.542 |
| Pollination efficiency index | 158.42±7.35 | 173.37±10.32 | -1.367 | 0.199 |
| Percentage of fruit set | 94.8718 | 97.3684 | -0.560 | 0.577 |
| Number of seeds/ fruit | 32.2973±0.9931 | 29.8108±1.325 | 1.501 | 0.138 |

**Table S4: Test of self-compatibility in *R. purpurea*.** Result of generalized linear model to examine the difference in fruit set percentage and seed number per fruit between hand-self and hand-cross pollinated flowers of *R. purpurea* at Daman and Mudhe.

|  | Fruit set percentage | | | Seed number per fruit | | |
| --- | --- | --- | --- | --- | --- | --- |
|  | df | deviance | P value | df | deviance | P value |
| Population (P) | 1 | 0.50566 | 0.783 | 1 | 2.9972 | 0.4300 |
| Treatment (T) | 1 | 0.06428 | 0.676 | 1 | 8.8733 | **0.0087** |
| P x T | 1 | 0.11215 | 0.738 | 1 | 0.4459 | 0.5043 |

**Table S5: Test of pollen limitation in *R. purpurea*.** Result of generalized linear model to examine the difference in fruit set percentage and seed number per fruit between natural and supplementary pollinated flowers of *R. purpurea* at two sites (Daman and Mudhe) and two years (2013 and 2014).

|  | Fruit set percentage | | | Seed number per fruit | | |
| --- | --- | --- | --- | --- | --- | --- |
|  | df | deviance | P value | df | deviance | P value |
| Year (Y) | 1 | 12.273 | **0.0004** | 1 | 0.0778 | 0.6449 |
| Population (P) | 1 | 0.001 | 0.4944 | 1 | 3.6774 | 0.0127 |
| Treatment (T) | 1 | 208.587 | **0.0000** | 1 | 4.3623 | 0.2010 |
| Y x P | 1 | 0.402 | 0.4590 | 1 | 1.7203 | 0.5451 |
| Y x T | 1 | 0.394 | 0.3835 | 1 | 0.0458 | 0.6077 |
| P x T | 1 | 0.000 | 0.6572 | 1 | 15.8415 | **0.0017** |
| Y x P x T | 1 | 0.357 | 0.5515 | 1 | 0.1873 | 0.6652 |

**
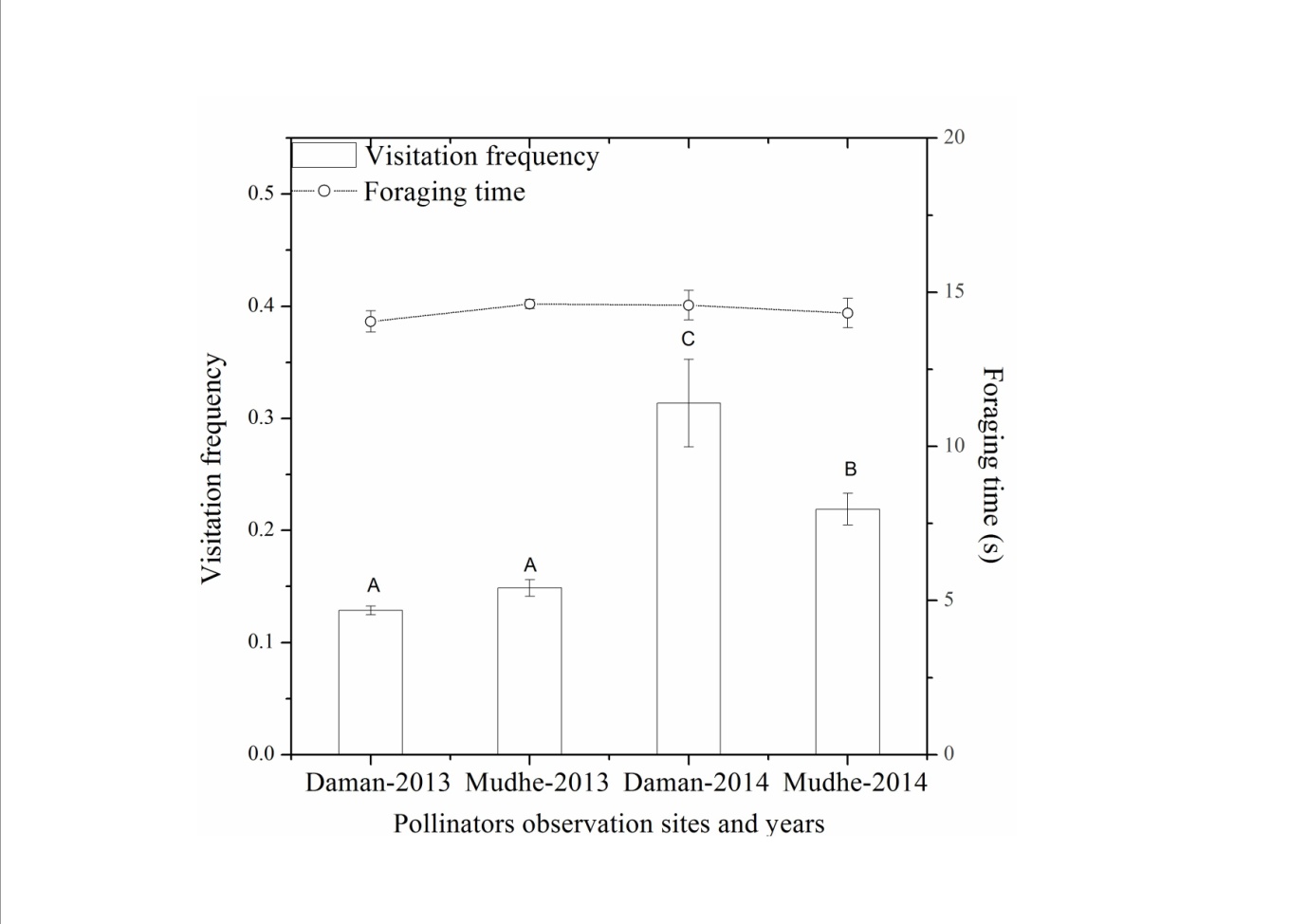
**

**Figure S1:** Variation in visitation frequency (visit/flower/hour) and foraging time (average time spent in a flower during a single visit) of *P. longirostris* in the flowers of *R. purpurea* between sites and years.

**
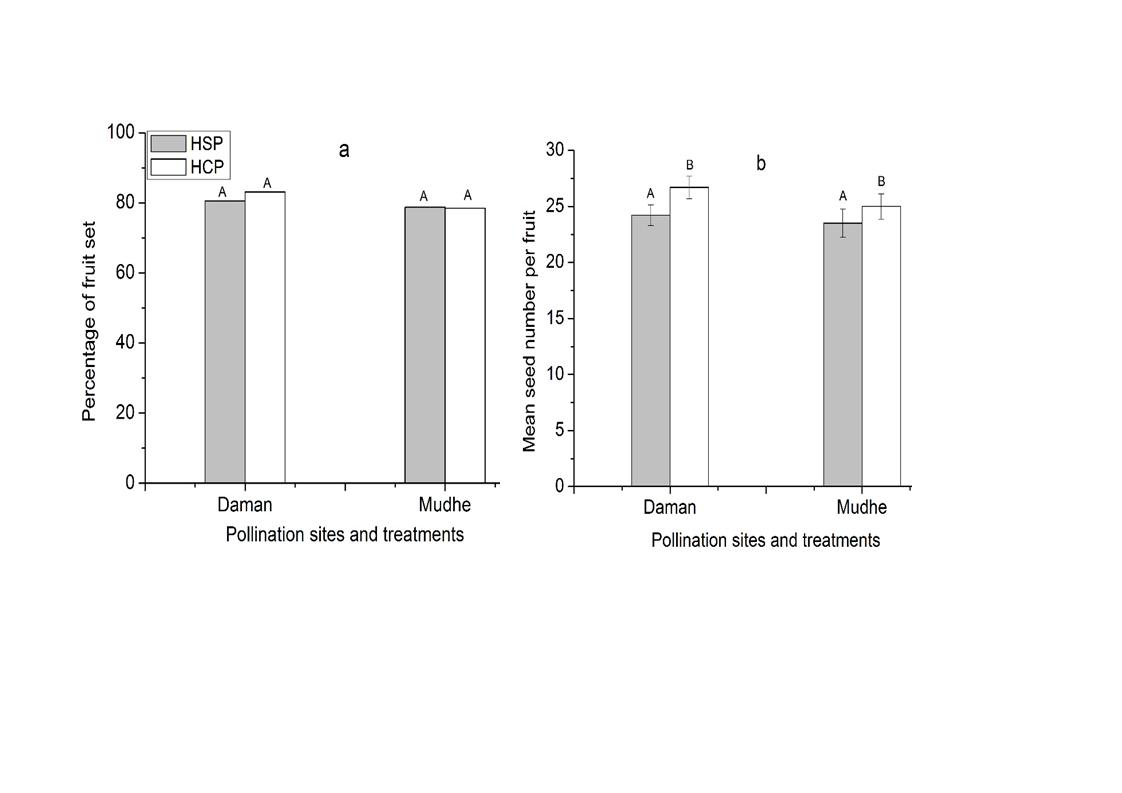
**

**Figure S2: Evidence of self-compatibility in *R. purpurea*:** Fruit set and seed set by hand self and hand cross pollination at two sites. a - Percentage of fruit set between hand self-pollination (HSP) and hand cross pollination (HCP) of *R. purpurea*. b- Number of seeds per fruit of HSP and HCP treatments. Error bars represent standard error. Different upper case letters indicate significant statistical difference at P<0.05.
